# P-1536. Prevalence and Characteristics of Escherichia coli Sequence Type 131 Isolated from Children with Urinary Tract Infections

**DOI:** 10.1093/ofid/ofaf695.1717

**Published:** 2026-01-11

**Authors:** Ji Young Park, Soyun Ahn

**Affiliations:** Department of Pediatrics, Seoul National University Children's Hospital, Seoul, Korea, Seoul, Seoul-t'ukpyolsi, Republic of Korea; Yeouido St. Mary’s Hospital, Seoul, Seoul-t'ukpyolsi, Republic of Korea

## Abstract

**Background:**

*Escherichia coli (E.coli)* is one of the most common causative pathogen of urinary tract infections in children. Antimicrobial resistance of *E. coli* has been and is continuing to increase globally. Sequence type (ST) 131 has emerged as a multidrug-resistant (MDR) pathogen. ST131 *E. coli* isolates have higher virulence than non-ST131 isolates. We aimed to characterize ST131 *E. coli* isolates from Korean children with UTIs compared to non-ST131 isolates and assess changes in their prevalence since 2011–2014.

**Figure:**
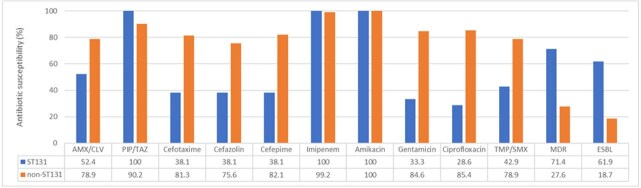
Antimicrobial susceptibility, ST131 vs. non-ST131 Escherichia coli ST131 isolates exhibited significantly lower susceptibility than non-ST131 isolates to amoxicillin/clavulanate, cefotaxime, cefazolin, cefepime, gentamicin, ciprofloxacin, and trimethoprim/sulfamethoxazole (all P<0.05). Susceptibility to piperacillin/tazobactam, imipenem, and amikacin remained comparable. ST131 isolates were significantly more likely to exhibit MDR and ESBL production.

**Figure:**
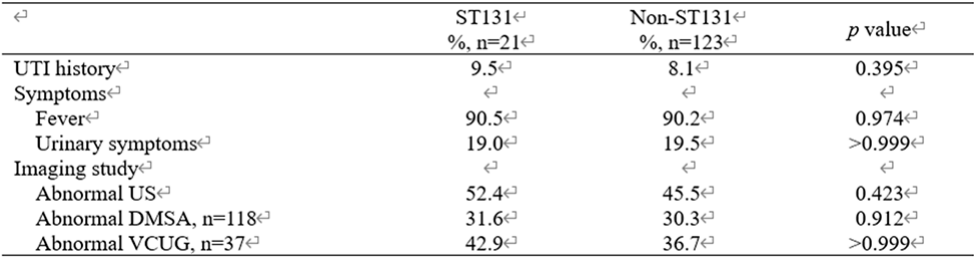
Clinical characteristics of patients infected with ST131 vs. non-ST131 Escherichia coli No significant differences were observed between the groups in prior UTI history or clinical symptoms such as fever and urinary symptoms. Also, imaging results, including ultrasonography, DMSA scans, and VCUG, did not differ significantly between groups.

**Methods:**

We analyzed the 144 culture-positive *E. coli* isolates and the medical records of the Chung-Ang University Hospital between January 2015 and June 2022. We Investigates the isolates of ST131 *E. coli* by using multilocus sequence typing.

**Results:**

A total of 144 *E. coli* isolates were analyzed, including 21 ST131 and 123 non-ST131 isolates. No significant differences were observed between the groups in prior UTI history or clinical symptoms such as fever and urinary symptoms. However, ST131 isolates exhibited significantly lower susceptibility (all *P*< 0.05) than non-ST131 isolates to amoxicillin/clavulanate (52.4 vs. 78.9%), cefotaxime (38.1 vs. 81.3%), cefazolin (38.1 vs. 75.6%), cefepime (38.1 vs. 82.1%), gentamicin (33.3 vs. 84.6%), ciprofloxacin (28.6 vs. 85.4%), and trimethoprim/sulfamethoxazole (42.9 vs. 78.9%). Susceptibility to piperacillin/tazobactam, imipenem, and amikacin remained comparable. ST131 isolates were significantly more likely to exhibit MDR and ESBL production (71.4 vs. 27.6% and 61.9 vs. 18.7%, all *P*< 0.05). Imaging results, including ultrasonography, DMSA scans, and VCUG, did not differ significantly between groups. Compared to a prior study at our center (2011–2014), ST131 prevalence slightly increased from 13.2% (15/114) to 14.6% (21/144), while ESBL-producing isolates rose markedly from 14.0% (16/114) to 25.0% (36/144). Notably, ST131 consistently demonstrated higher ESBL positivity than non-ST131 in both periods (53.3% vs. 8.1% in 2011–2014; 61.9% vs. 18.7% in 2015–2022), indicating a persistent resistance trend.

**Conclusion:**

ST131 *E. coli* isolates in children with UTIs exhibited higher rates of MDR and ESBL production and their prevalence has slightly increased over time, highlighting the need for continued surveillance.

**Disclosures:**

All Authors: No reported disclosures

